# Hidden Vibrational Bistability Revealed by Intrinsic Fluctuations of a Carbon Nanotube

**DOI:** 10.1021/acs.nanolett.4c06618

**Published:** 2025-04-29

**Authors:** P. Belardinelli, W. Yang, A. Bachtold, M. I. Dykman, F. Alijani

**Affiliations:** † Department of Construction, Civil Engineering and Architecture, Polytechnic University of Marche, 60131 Ancona, Italy; ‡ 172281 ICFO - Institut de Ciencies Fotoniques, The Barcelona Institute of Science and Technology, 08860 Castelldefels, Barcelona, Spain; § Department of Physics and Astronomy, 3078 Michigan State University, East Lansing, Michigan 48824, United States; ∥ Department of Precision and Microsystems Engineering, Delft University of Technology, Mekelweg 2, 2628CD Delft, Netherlands

**Keywords:** carbon nanotube, self-oscillations, hysteresis-free bistability, stochastic switching, nonlinear friction

## Abstract

We demonstrate that a quiet state and large-amplitude self-sustained oscillations can coexist in a carbon nanotube subject to time-independent drive. A feature of the bistability is that it would be hysteresis free in the absence of noise, and the oscillatory state would not be seen. It is revealed by random switching between the stable states, which we observe in the time domain. We attribute the switching to fluctuations in the system and show that it displays Poisson statistics. We propose a minimalistic model that relates the emergence of the bistability to a nonmonotonic variation of nonlinear friction with the vibration amplitude. This new type of dynamical regime and the means to reveal it are generic and are of interest for various mesoscopic vibrational systems.

## Graphical abstract

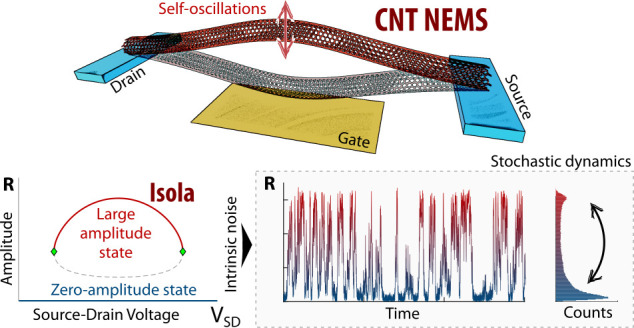

Nanoelectro-mechanical systems (NEMS) provide a means for studying physics away from thermal equilibrium in a well-characterized setting.^1^ An important group of nonequilibrium phenomena originates from the interplay between nonlinearity and fluctuations in driven systems, which can modify the frequency stability,^2–6^ the power spectrum,^7,8^ lead to spectral broadening,^9^ and thermal noise squeezing.^8,10,11^ The interplay is most nontrivial when a nonequilibrium system is brought into a regime where it exhibits bistability. Here, fluctuations, even if weak on average, can cause interstate transitions and are ultimately responsible for the distribution of a system over the stable states.^1^ Much work on studying these effects and the emerging scaling^12,13^ has been carried out on nano- and micromechanical resonators driven by an external resonant force or modulated parametrically.^7,14–23^ A mechanism that leads to the onset of bistability in NEMS without periodic driving was suggested in ref^24^ and such bistability was observed in a carbon nanotube (CNT).^25^

In almost all bistable mesoscopic vibrational systems, the vibrations could be brought to one of the stable states by smoothly changing a control parameter, for example, the driving force. As a result of the change, at some critical parameter value, the bifurcation point, one of the stable states would lose stability, and the system would switch to another stable state. Such behavior is usually accompanied by hysteresis: in a parameter range between bifurcation points the state of the system depends on the history of the parameter change. In all works on NEMS thus far hysteresis was used to reveal the bistability.

In this paper, we report the observation of a hysteresis-free bistability in a nanomechanical system. Such bistability means that, as the control parameter is changed back and forth, the system remains in the quiet state. The very presence of another stable state is revealed by fluctuations that cause interstate transitions. In our system, coexisting are the quiet state and the state of large-amplitude self-sustained oscillations of the lowest mode of a CNT driven by a time-independent source-drain voltage, see Figure 1. The large-amplitude oscillatory state was already identified in ref 26. It is important that the mode experiences fluctuations. We reveal that the system is actually bistable by examining the fluctuation statistics. In distinction from the more conventional scenario, the large-amplitude oscillatory state emerges as the source-drain voltage is increased, and when it emerges, it is already well separated from the quiet state in phase space. Then, as the source-drain voltage is further changed, the vibrational state is observed to lose stability. There is no hysteresis when the bifurcation parameter is moved back and forth. In terms of the bifurcation theory,^27^ the emerging and disappearing stable states are associated with an “isola”: an isolated branch of an equilibrium state of a noise-free system.

**Figure 1:**
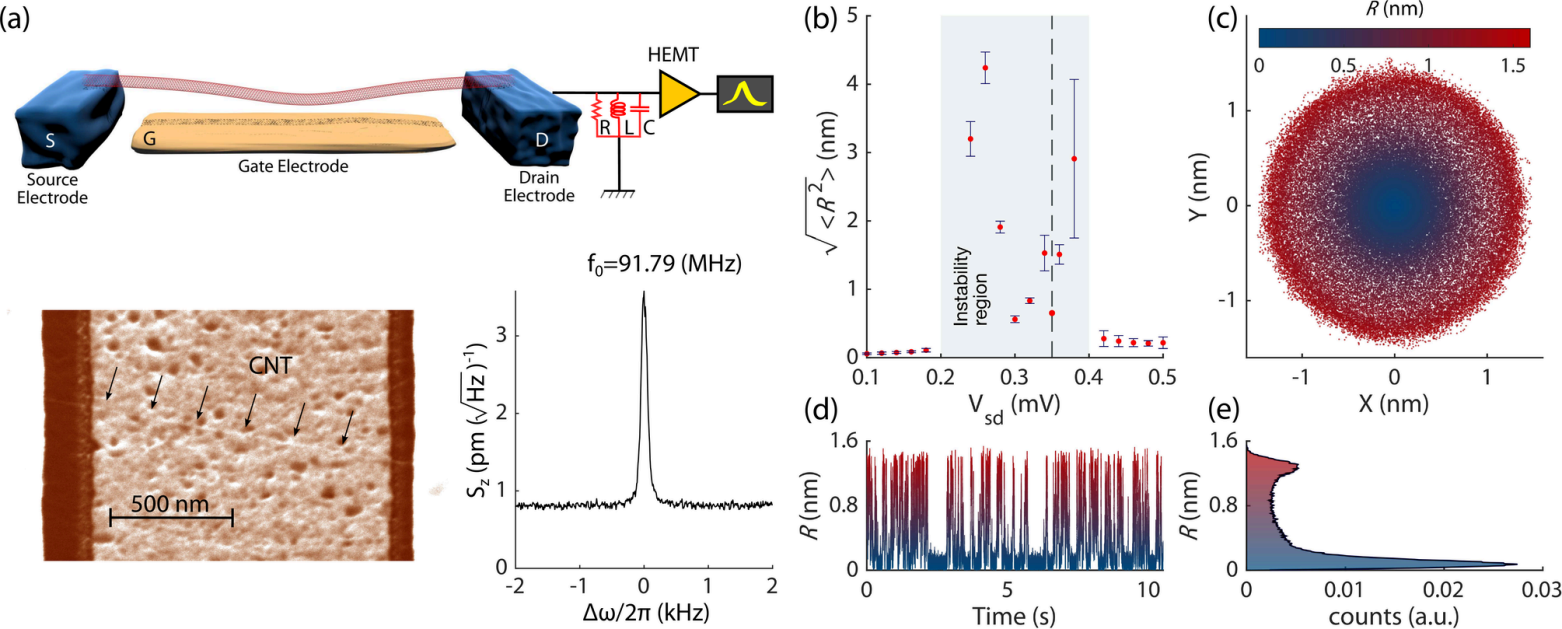
(a) CNT-based electromechanical oscillator and measurement schematic. The CNT has a length of ≈1.5 μm and a radius of ≈1 nm (scanning electron microscope image in the bottom left panel). Voltages *V*_sd_ and *V*_g_ are applied to electrodes S and Gate, respectively. The drain electrode D is connected to an RLC resonator (*f*_RLC_ = 1.27 MHz). The displacement spectral density is shown in the bottom right panel. (b) The root-mean-square nanotube displacement from the origin $\sqrt{\left\langle R^{2}\right\rangle }$ as a function of the source-drain voltage *V*_sd_ for *V*_g_ = −616 mV; $\sqrt{\left\langle R^{2}\right\rangle }$ is obtained from spectral noise measurements for all the data points except for *V*_sd_ = 0.35 mV (dashed line). The latter is obtained by recording the time evolution of the displacement amplitude at the mode antinode. (c) The measured two quadratures X and Y of the motion on the (X, Y)-plane at this *V*_sd_. (d) Fluctuations of the amplitude $R=\sqrt{X^{2}+Y^{2}}$ in time for the same *V*_sd_. (e) Amplitude histogram, normalized with respect to the total number of observations.

The experiment is done using clamped–clamped CNTs grown by chemical vapor deposition across two metallic contact electrodes.^28^ The measurements are performed at cryogenic temperature (70mK) by applying a voltage bias to the source (*V*_sd_) and to the gate electrode (*V*_*g*_) which is placed beneath the CNT. The current from the drain electrode is measured by using a RLC resonant circuit and a low-temperature HEMT amplifier, see Figure 1(a). The read-out signal is obtained by measuring the current noise spectrum, which is converted to the nanotube displacement.^26^ The same calibration is used to obtain the quadratures of the motion (X,Y) from the lock-in measurements. The scaled vibration amplitude is $R=\sqrt{X^{2}+Y^{2}}$ .

When sweeping up the source-drain voltage *V*_sd_ at *V*_g_ = −616 mV, there occurs a sudden jump up in the displacement *R* for *V*_sd_ ≈ 0.2 mV followed by a jump down at *V*_sd_ ≈ 0.4 mV, see highlighted region of Figure 1(b). The change of the nanotube motion with *V*_sd_ in Figure 1(b) does not resemble that of a vibrational system undergoing a supercritical Hopf bifurcation, whose signature is a smooth monotonic increment of the oscillation amplitude and loss of stability of the quiet state.^29^ The instability is observed over a narrow range of gate voltage *V*_g_ but reappears periodically in *V*_g_ with a period corresponding to adding four electrons to the nanotube. The periodicity is consistent with the SU(4) symmetry of the CNT.^26^

In the (X,Y) space at *V*_sd_ = 0.35 mV we recognize a highly populated doughnut-like region centered at a nonzero mean amplitude that encircles the thermal motion about the origin of the quadrature space. This region suggests the presence of an oscillatory state, see Figure 1(c). The time trace of the motion (Figure 1(d)) and the normalized histogram of the amplitude *R* in Figure 1(e) further support the notion that the system has two different dynamical states. While distinct peaks in the amplitude distribution may serve as a signature of coexisting states, a double-peak pattern can also arise in various other dynamical phenomena such as intermittent chaos or bursting oscillations.^30^ A careful analysis is required to differentiate bistability from aperiodic dynamical behaviors.

To understand the nature of the observed dynamics, we perform statistical analysis on the time-domain data of Figure 1(d). This data set has not been presented in the earlier work.^26^ We assume that our system has two stable states, namely a zero-amplitude, i.e. a quite state, and a self-sustained oscillatory state, which has a large amplitude compared to the root-mean-square amplitude fluctuations.^31–34^ We investigate whether noise induces stochastic transitions between the stable states, analogous to the noise-induced interwell hopping of a damped particle in a double-well potential, see Figure 2(a), cf.;^35^ such hopping underlies stochastic resonance.^36,37^ The difference in our case is that here one of the stable states is a static equilibrium point, whereas the other is a state of self-sustained vibrations.

**Figure 2:**
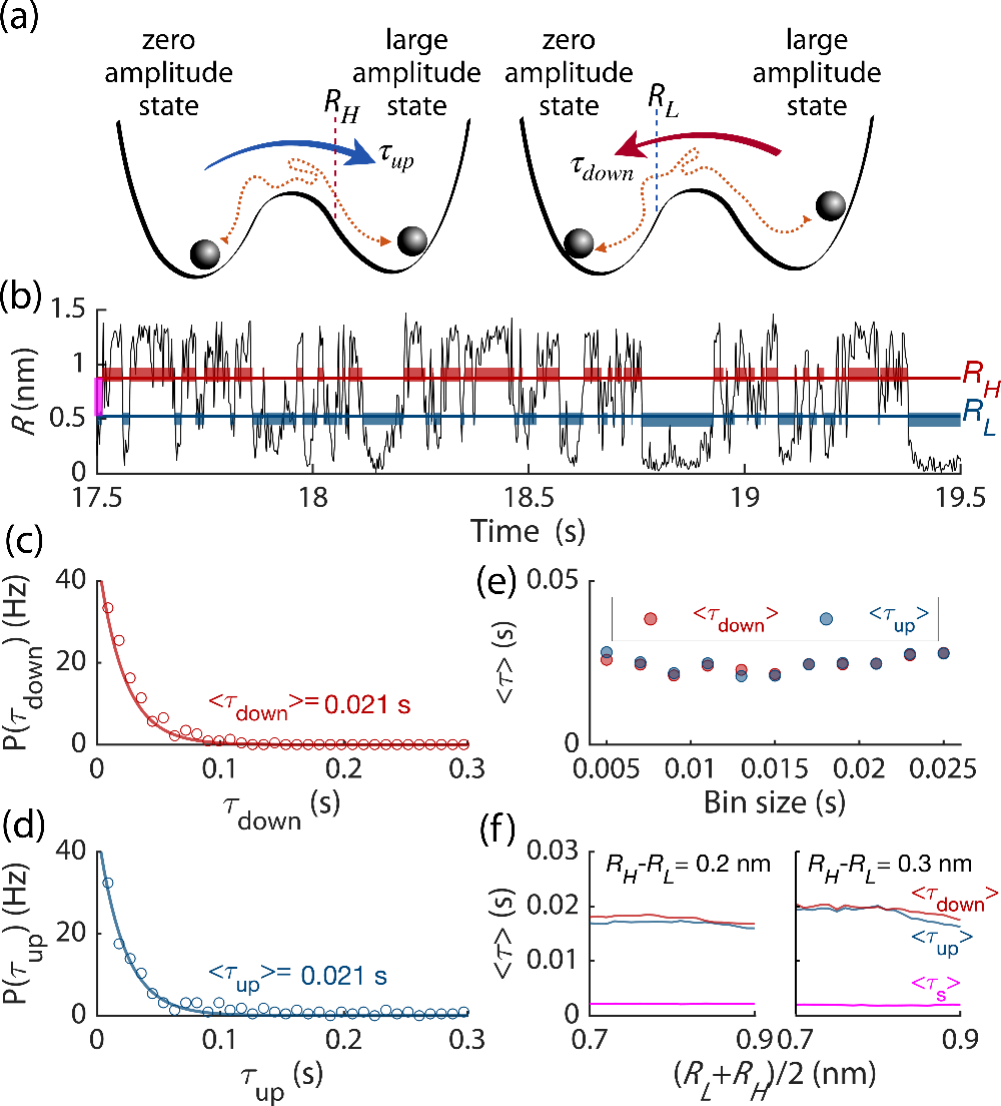
(a) A double-well potential, illustrating bistable dynamics using a ball-in-a-cup analogy. The minima are associated with the stable state of self-sustained vibrations and the zero-amplitude state of the CNT. Noise-induced transitions to the large-amplitude (zero-amplitude) states are considered to occur once the vibration amplitude crosses the threshold *R*_*H*_(*R*_*L*_). (b) A sample of the time evolution of the vibration amplitude (*V*_sd_ = 0.35 mV). Blue/red bars indicate the chosen switching thresholds with R_*H*_ – R_*L*_ = 0.35 nm, (R_*H*_ + R_*L*_)/2 = 0.7 nm. The magenta bar indicates the region between *R*_*H*_ and *R*_*L*_. (c, d) Dwell (residence) time distributions (bin size 9 ms) for the large-amplitude state [panel (c)] and the zero-amplitude state [panel (d)]. A Poisson distribution (eq 1) is fitted to the data. It gives averaged dwell times of ⟨τ_down_⟩ = 0.021 s and ⟨τ_up_⟩ = 0.021 s. (e) Influence of the bin size on the average dwell times in panels c and d. (f) Average dwell times for varying thresholds *R*_*L*_ and *R*_*H*_. The average time ⟨τ_*s*_⟩ is the time spent in between the two thresholds.

Noise-induced switching is well-defined if the switching rate is much smaller than the relaxation rate. A noise-driven system then spends most of the time fluctuating about one of its stable states. The characteristic correlation time *t*_*r*_ of these fluctuations is the dynamical relaxation time or the correlation time of the noise. Occasionally there occur large outbursts of noise that lead to switching between the states. The typical time between such outbursts is much larger than *t*_*r*_, whereas the duration of the switching event itself is comparable to *t*_*r*_. Therefore, the switching events are expected to be uncorrelated and described by the Poissonian statistics.

In order to detect interstate switching events, one would need to set a threshold, which is related to but does not coincide with the basin boundary of the two states. For a particle in a double-well potential, reaching the barrier top does not necessarily lead to switching, as the system can go back to the initially occupied well. Even in the simplest case of fluctuations induced by white noise, to find the switching rate one has to set up a threshold sufficiently far beyond the barrier top with respect to the initially occupied state, as has been known since the classical work of Kramers.^38^ It is clear from the above arguments that the thresholds for transitions between different states should not coincide, cf.^39^ Hence, we introduce two amplitude thresholds, *R*_*L*_ and *R*_*H*_, see Figure 2(a). If the system was fluctuating about R = 0 and its trajectory crossed *R*_*H*_, we assume that it has switched to the large-amplitude state. On the other hand, if the system was fluctuating about the large-amplitude state and its trajectory *R*(*t*) crossed *R*_*L*_, it has switched to the R = 0 state. With the above definition, the dwell (residence) times τ_up_ and τ_down_ are the times spent in the zero- and large-amplitude states, respectively, before the system switches. The time intervals τ_up_ and τ_down_ are shown in Figure 2(b) by the red and blue thick bars, respectively. The region between *R*_*H*_ and *R*_*L*_ contains the separatrix. The experimental data do not allow us to find it.

Figures 2(c) and 2(d) show the distribution of the dwell times τ_up_ and τ_down_. The transitions between the states are well described by a Poisson process, and the distribution of the dwell times is close to exponential,

1 $$P\left(\tau \right)=\frac{1}{\left\langle \tau \right\rangle }e^{-\tau /<\tau >}$$

This is the central argument in support of the coexistence of two stable states in our system. We use eq 1 to fit the experimental data and find that the dwell times are approximately the same for the chosen parameters, with τ_up_ ≈ τ_down_ ≈ 21 ms. The fit is only mildly influenced by the bin size, see Figure 2(e). These dwell times are much larger than the relaxation time *t*_*r*_ of the nanotube, which can be inferred from duration of the switching events themselves: in Figure 2(b) the trajectories leading to transitions are essentially vertical. The detailed data indicates that *t*_*r*_ ∼ 1–3 ms, as illustrated in Figure 2(f) by the average time ⟨τ_*s*_⟩ spent in between the two thresholds.

Another important argument in support of the bistability is seen from Figure 2(f). In this figure we plot the dwell times over a broad range of mean threshold values and separations. The results do not change. This demonstrates the reliability and stability of the two-threshold approach and confirms the presence of noise-induced hopping between two metastable states. The stochastic analysis conducted on an additional temporal data set, corresponding to *V*_sd_ = 0.25 mV, also aligns with these findings (see Supporting Information S1 ^40^).

The bistable dynamics observed in Figure 2 ultimately comes from the source-drain voltage *V*_sd_, which pumps energy into the system. The onset of self-sustained vibrations due to energy pumping is often associated with the friction coefficient becoming negative, which makes the quiet state unstable. In contrast, in our system the quiet state remains stable. This can be understood if the friction coefficient becomes negative in a certain range of sufficiently large vibration amplitude. The dependence of the friction coefficient on amplitude is called nonlinear friction. Such friction is well-known in nanomechanics.^1,41^ Usually it leads to a faster decay of vibrations with the increasing amplitude, that is, the coefficient of nonlinear friction is positive, although there has been also observed slowing down of the decay with the increasing amplitude.^42^

There are several possible causes of nonlinear friction in our system. One of them is the electron-vibrational coupling. As electrons hop between the leads and the nanoresonator, they exchange energy with the mode. This leads to decay or excitation of the vibrations, i.e., to positive or negative friction, see^24,43–47^ and references therein. The analyses in these papers refer to the limit of strong Coulomb blockade. Negative nonlinear friction resulted from the dependence of the tunneling on the vibration amplitude.^24,45^ This dependence should occur in our system, too, even though the Coulomb gap is moderately hard. One can picture this dependence as coming from the change of the transmission of the tunneling barrier due to the strain induced by the CNT displacement.^48^

In the basic model of the effect of vibrations on tunneling^49^ the vibration-induced change of the tunneling exponent is *C*_tun_ *q*/λ_tun_, where *q* is the mode coordinate and λ_tun_ is the electron tunneling length. In the measurements presented in this work, the tunnel barriers are defined in the clamping areas at the interface between the nanotube and the metal electrodes. For tunneling onto/from a CNT, the coefficient *C*_tun_ depends on the structure of this interface, which is not well characterized, and therefore it cannot be found quantitatively. However, the ratio *q*/λ_tun_ itself is ≳ 10 for the observed limit cycle radius and λ_tun_ ∼ 3–4 Å. This suggests that the friction that results from the modulation of the tunneling barrier can be significantly nonlinear. It can be negative in the range of *V*_sd_ where the energy transfer to the mode exceeds the energy drain from the mode. The amplitude dependence of the friction is affected also by the polaronic effect: because of the gate voltage, electrons exert force on the mode that depends on the number of electrons on the CNT, which itself depends on the CNT displacement. This force leads to vibration decay, for strong Coulomb blockade.^46^

Another source of negative nonlinear friction, a retarded backaction from the circuit, is discussed in the SI Sec. S2.^40^ Here, a quantitative theory requires the knowledge of the nonlinear dependence of the conductance on the vibration amplitude, which itself requires full characterization of the clamping area. However, the magnitude of this nonlinear friction force compared to the linear one is proportional to the square of the ratio of the displacement amplitude to the distance to the gate electrode and thus relatively small.

The above arguments show that the situation with nonlinear friction is no different from linear friction, which comes from several possible mechanisms. Therefore, to describe the central experimental observation, the onset and collapse of self-sustained vibrations, we use a minimalistic model. A major feature of this model is the absence of delay in the friction force, in the rotating frame. This is a consequence of the smoothness of the density of states of the electrons and thermal acoustic phonons involved in the mode decay processes, cf.^1^

Since the vibration frequency is much higher than all other rates and frequencies in the system, the vibrations can be described in the rotating frame using a complex vibration amplitude *z*(*t*) = *C*_*z*_[*q* + *i*(*p*/*mω*_0_)] exp­(*iω*_0_ *t*), where *q* and *p* are the mode coordinate and momentum, *m* is its effective mass, ω_0_ is the eigenfrequency and *C*_*z*_ is a scaling parameter. In the rotating wave approximation (RWA) the equation of motion (see Sec. S3 of the Supporting Information^40^) reads

2 $$ż=-\left[\Gamma +\left(\gamma_{nlf}-i\gamma_{D}\right){\left|z\right|}^{2}+{\left|z\right|}^{4}\right]z$$

Here Γ and γ_nlf_ are the coefficients of linear and nonlinear friction, whereas γ_*D*_ is the Duffing nonlinearity. The RWA applies provided $\left|ż\right|\ll \omega_{0}\left|z\right|$ . The right-hand side of eq 2 is our minimalistic model of nonlinear friction: it is an expansion in *z*, valid when the vibration amplitude is comparatively small, so that the decay rate and the change of the vibration frequency are ≪ ω_0_. The term ∝|*z*|^4^ *z* describes quintic nonlinear friction, cf.^50^ It must be taken into account where the conventional friction coefficients Γ and γ_nlf_ become small. A simple microscopic model of such friction is provided in Supporting Information S4.^40^ In distinction from the conventional analysis of the onset of self-oscillations, which uses the model (2) without the quintic term, to describe the experiment we have to assume that the parameter Γ remains positive, and it is γ_nlf_ that is the bifurcation parameter that changes sign.

We rewrite eq 2 in polar coordinates by setting *z*(*t*) = *R*(*t*)*e* ^*iθ*(*t*)^, where *R* and θ are the vibration amplitude and the “slow” part of the vibration phase. From eq 2

3 $$Ṙ=-f_{nlf}R,,\mathrm{\theta ̇}=\gamma_{D}R^{2}$$

with the coefficient of nonlinear friction being

4 $$f_{nlf}=\Gamma +\gamma_{nlf}R^{2}+R^{4}$$

For Γ > 0 the quiet state R = 0 is stable. If Γ becomes negative, the state R = 0 becomes unstable, and for γ_nlf_ > 0 there emerges a stable limit cycle with radius $\propto {\left(\left|\Gamma \right|/\gamma_{nlf}\right)}^{1/2}$ (supercritical Hopf bifurcation). If Γ is positive but γ_nlf_ becomes negative, there emerges an unstable limit cycle with radius ${\left(\Gamma /\left|\gamma_{nlf}\right|\right)}^{1/2}$ (subcritical Hopf bifurcation).

The term *R* ^4^ in *f*_nlf_ leads to the onset of a stable limit cycle for γ_nlf_ < 0 and Γ > 0 along with an unstable one. The radii of the cycles as given by the condition *f*_nlf_ = 0 are

5 $$R_{\pm }=\frac{1}{\sqrt{2}}{\left[-\gamma_{nlf}\pm \sqrt{\gamma_{nlf}^{2}-4\Gamma }\right]}^{1/2}$$

For −γ_nlf_ > 2Γ^1/2^ the cycle with the radius *R*_+_ is stable, whereas the cycle with the radius *R*_–_ is unstable. At γ_nlf_ = −2Γ^1/2^ the two cycles merge and annihilate one another in a saddle-node bifurcation. For smaller |γ_nlf_|/2Γ^1/2^ they disappear.

We now relate model (2) to the experiment. The values of Γ and γ_nlf_ change with the control parameter *V*_sd_. In particular, Γ decreases as we approach the bistability region in Figure 1(b).^26^ The key observations are (i) a stable zero-amplitude state and a stable limit cycle coexist in a certain parameter range, (ii) the zero-amplitude state is stable not only outside, but also inside this range, and (iii) the limit cycle is excited and collapses with the varying parameters while still having a large amplitude. This scenario is qualitatively different from the standard subcritical Hopf bifurcation that is accompanied by hysteresis.

A minimalistic picture that describes the experiment is that, as the source-drain voltage *V*_sd_ varies, there first occurs a saddle-node bifurcation at −γ_nlf_ = 2Γ^1/2^. At this bifurcation there emerge the stable and unstable limit cycle with amplitudes *R*_±_. As *V*_sd_ varies further, these limit cycles merge together and disappear via another saddle-node bifurcation. This is illustrated in Figure 3.

**Figure 3:**
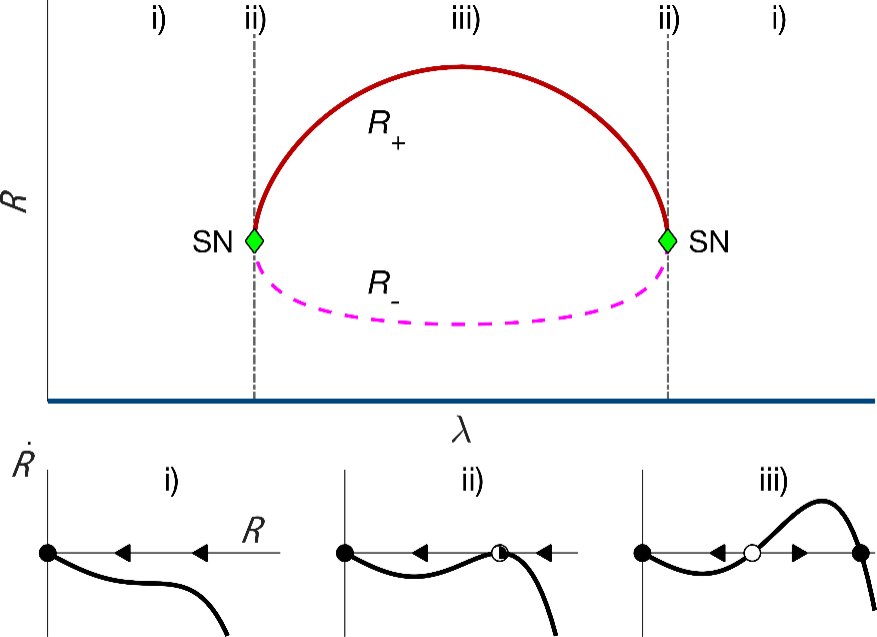
(Upper panel) Steady-state solutions as a function of the bifurcation parameter λ. In our nanomechanical resonator λ is a function of *V*_sd_. Solid/dashed lines are stable (*R*_+_)/unstable (*R*_–_) solution branches. The isola emerges as a result of nonmonotonic nonlinear damping. (Lower panel) Sketches of the phase portrait of the vibration radius. (i) The radial phase portrait for λ below and above the isola bifurcation point. The solid dot is a stable solution. (ii) The saddle-node bifurcation (SN) at a finite vibration radius, indicating the onset of an isola. (iii) Bistable region with coexisting zero and large amplitude states. The open circle is an unstable solution, which corresponds to an unstable limit cycle. The arrows in panels (i), (ii), and (iii) indicate the dynamical flow.

To link the above phenomenology to eqs 3 and 4, we should consider how the parameters of these equations depend on *V*_sd_. A major factor is the *V*_sd_-dependence of γ_nlf_, since this parameter becomes negative and, moreover, exceeds 2Γ^1/2^ in the absolute value. It should be noted that the linear friction coefficient Γ also depends on *V*_sd_, as reported in.^26^ Overall, γ_nlf_ should be nonmonotonic as a function of *V*_sd_ to allow for both the onset and the disappearance of the bistability with the increasing *V*_sd_. The analysis simplifies for the gate voltage *V*_*g*_ where the range of *V*_sd_ in which the zero-amplitude state coexists with the vibrational state is narrow. In this case one can approximate

6 $$\gamma_{nlf}\left(V_{sd}\right)+2\sqrt{\Gamma \left(V_{sd}\right)}=-\eta \left(V_{B}^{\left(1\right)}-V_{sd}\right)\left(V_{sd}-V_{B}^{\left(2\right)}\right)$$

Here, the parameter η > 0 is a scaling parameter and *V*_*B*_^(1,2)^ are the bifurcational values of *V*_sd_. The CNT shows bistabilty in the range *V*_*B*_^(2)^ < *V*_sd_ < *V*_*B*_^(1)^.

The quadratic dependence of γ_nlf_ and the corresponding dependence of *R*_+_ on *V*_sd_ provide an insight into our observations, but do not fully describe the evolution of the dynamics within the instability region. The complicated dependence of *R*_+_ on *V*_sd_ can have several causes, including defects in the CNT that lead to a nonuniform electron density, as well as the interplay of the Kondo effect and the Coulomb blockade, which depend on the bias and the gate voltage, thus modifying the tunneling and the polaronic effect and ultimately the friction force. In Figure 4 we show that the complex behavior of the vibration amplitude within the instability region can be effectively captured by considering a nonmonotonic dependence of the nonlinear friction force on the source-drain voltage. Given that the dependence of the root-mean-square vibration amplitude ${\left\langle R^{2}\right\rangle }^{1/2}$ on *V*_sd_ in Figure 1(b) resembles an inverted quartic parabola, we describe the dynamics within the bistability region by a constant linear friction and a 5-parameter nonlinear friction, γ_nlf_=∑_*n* = 0_^4^(γ_*n*_ *V*_sd_^*n*^). To numerically obtain the parameters that match the experiment we positioned the saddle-node points of the isola at the boundaries of the instability region observed experimentally and constrained the amplitude of the self-oscillations to match the measured $\sqrt{\left\langle R^{2}\right\rangle }$ values.

**Figure 4:**
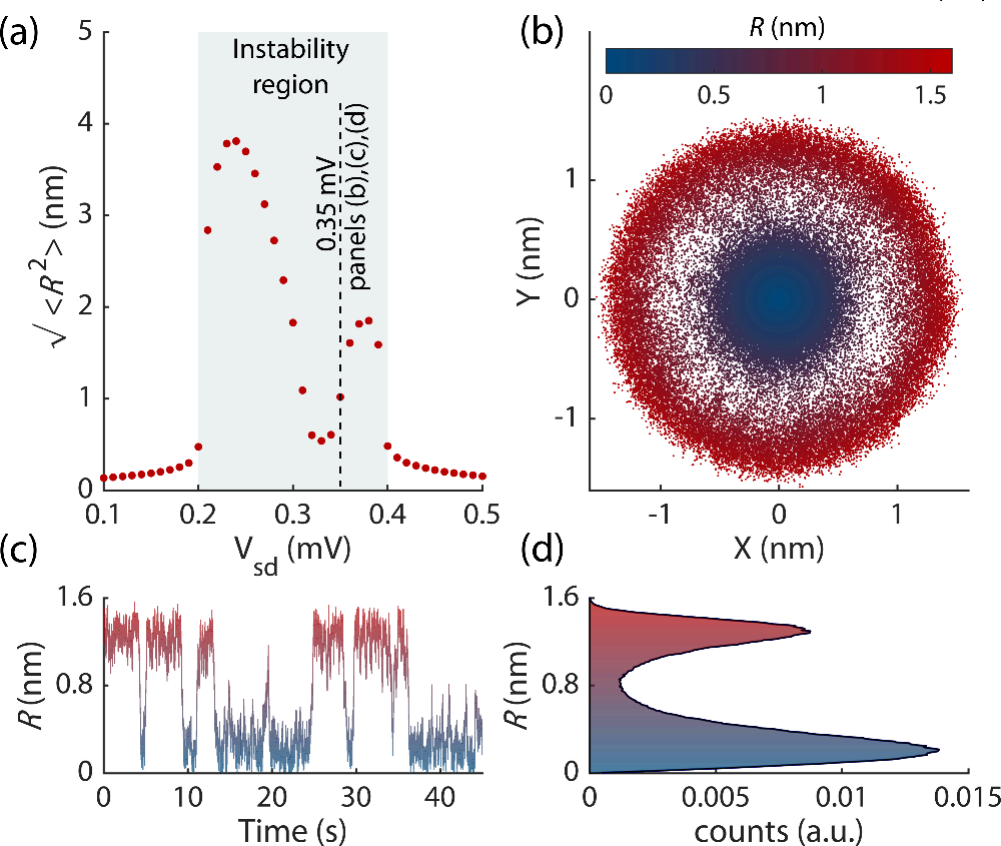
Numerical simulations of the fluctuation dynamics of the nanotube. (a) The standard deviation of the nanotube displacement $\sqrt{\left\langle R^{2}\right\rangle }$ as a function of the source-drain voltage *V*_sd_. Each point is the average of ten simulations. The stochastic dynamics is characterized for one simulation at *V*_sd_ = 0.35 mV (dashed line) in panels (b–d). (b) The phase space of the two quadratures of the motion (X,Y). (c) Fluctuations of the amplitude $R=\sqrt{X^{2}+Y^{2}}$ in time. (d) The amplitude histogram of the time trace of panel (c) normalized with respect to the total number of observations. Details of the simulations of the stochastic dynamics of the nanotube are provided in Sec. S5 of the Supporting Information.^40^

We plot in Figure 4(a) the evolution of the amplitude variance as a function of *V*_sd_ by simulating the stochastic dynamics of eq 2 in the quadrature space, in which each quadrature is affected by an independent random Wiener process (See Sec. S5 of the Supporting Information^40^ for more details). The noise, which is white in the slow time $∼\Gamma^{-1},{\left(\left|\gamma_{nlf}\right|\left\langle R^{2}\right\rangle \right)}^{-1}$ , comes from different intrinsic sources, such as hopping of the electrons on and off the CNT and creation and annihilation of thermal phonons nonlinearly coupled to the mode. In the phase-space of the two quadratures (X,Y) the trajectories fluctuate about a circle with radius ${\left\langle R^{2}\right\rangle }^{1/2}$ or about the quiet state *R=0*, switching between them, see Figure 4(b) and (c). Respectively, the stationary probability distribution of *R*(*t*) displays two peaks, as seen in Figure 4(d).

We emphasize that the nonmonotonic dependence of the nonlinear friction parameter γ_nlf_ on *V*_sd_, with γ_nlf_ being negative in a certain range of *V*_sd_, is critical for the emergence of the isola and the hysteresis-free bistability. At the same time, fluctuations in the system are crucial for revealing the bistability.

Although isolas in multistable systems have attracted much attention theoretically,^51–54^ their experimental demonstrations have almost exclusively been limited to macroscale systems under periodic driving.^55–57^ In mesoscopic systems, the only reported observation of isolas involves forced vibrations of a nonlinear microresonator with coupled vibrational modes.^58^ In nanomechanis, isolas have not been observed. Here, we demonstrate the existence of an isolated vibrational state in a nanomechanical system subject to a time-independent drive. Specifically, we show that driving a carbon nanotube by a dc source-drain voltage *V*_sd_ leads to the onset of bistability, in which a stable quiet state coexists with periodic self-sustained vibrations. We show that the bistability is nonhysteretic: varying the control parameter would not lead to switching between the branches of the stable states. We also provide a minimalistic phenomenological model that describes the effect and indicate the mechanisms that can underlie this model.

## Supplementary Material



## Notes

Additional analysis of statistics of stochastic switching of the carbon nanotube, discussion on friction force generated due to electrothermal backaction, theoretical description of mechanism leading to quintic nonlinear friction, and more information on numerical simulations (PDF)
