# Supplementary material for: Hidden Vibrational Bistability Revealed by Intrinsic Fluctuations of a Carbon Nanotube
Source: Nano Lett. 2025 Apr 29;25(21):8443–9. doi: 10.1021/acs.nanolett.4c06618 (PMC12123673; doi:10.1021/acs.nanolett.4c06618)
Supplement: Supplementary file 1 [file nl4c06618_si_001.pdf]

Supporting Information: Hidden vibrational bistability revealed by intrinsic fluctuations of a carbon nanotube

P. Belardinelli,[†] W. Yang,[‡] A. Bachtold,[*,‡] M.I. Dykman,[*,¶] and F. Alijani[*,§]

†*Department of Construction, Civil Engineering and Architecture, Polytechnic University of Marche, Ancona, Italy*

‡*ICFO - Institut de Ciencies Fotoniques, The Barcelona Institute of Science and Technology, 08860 Castelldefels, Barcelona, Spain*

¶*Department of Physics and Astronomy, Michigan State University, East Lansing, MI 48824, USA*

§*Department of Precision and Microsystems Engineering, Delft University of Technology, Mekelweg 2, 2628CD, Delft*

E-mail: Adrian.Bachtold@icfo.eu; dykmanm@msu.edu; f.alijani@tudelft.nl

S1. Additional data on stochastic switching in the carbon nanotube

In order to demonstrate the repeatability of switching behavior within the bistable region, here we analyze an additional dataset (V_{sd} =0.25 mV and V_{g} =−616 mV). The findings from this supplementary data shown in Fig. S1 are consistent with our results at V_{sd} =0.35 mV and V_{g} =−616 mV (Fig. 2 of the main manuscript). To summarize, we report: *i)* comparable shape of the dwell (residence) time distributions (panels c-d); *ii)* mild influence of the bin

size on the statistics and *iii)* dwell times unchanged over a broad range of mean threshold values and separations.

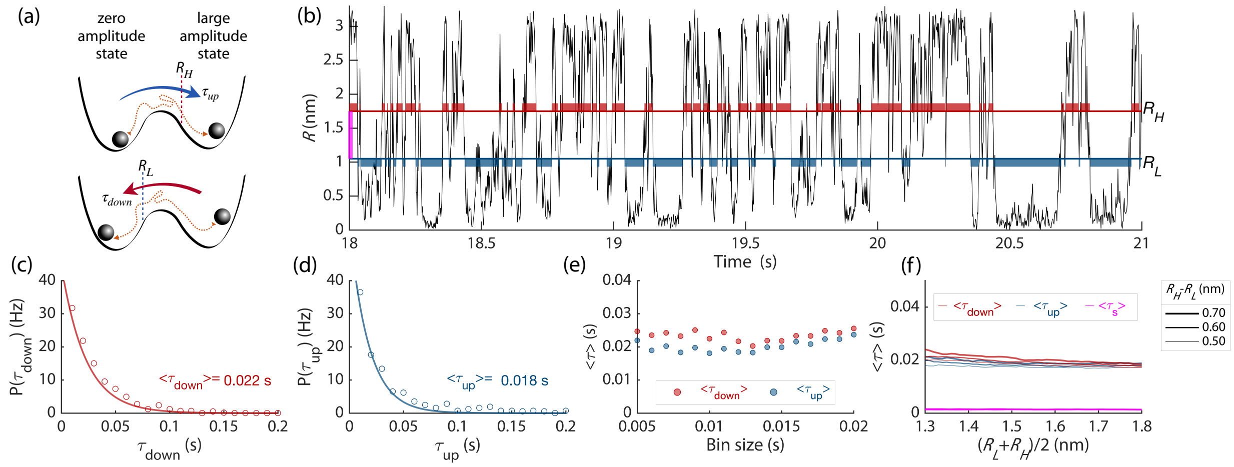

Fig. S1: (a) Sketch of a bistable potential. The minima are associated with the stable states of self-sustained vibrations and the zero-amplitude state of the CNT. Noise-induced transitions to the large-amplitude(zero-amplitude) states are considered to occur once the vibration amplitude crosses the threshold $R_H(R_L)$. (b) A sample of the time evolution of the vibration amplitude (V_{sd} =0.25 mV). Blue/red bars indicate the chosen switching thresholds with R_H-R_L=0.7nm, $(\mathrm{R}_H+\mathrm{R}_L)/2$=1.4nm. The magenta bar indicates the region between R_H and R_L. (c)-(d) Dwell (residence) time distributions (bin size 10 ms) for the large-amplitude state [panel (c)] and the zero-amplitude state [panel (d)]. A Poisson distribution (Eq. 1 of the main manuscript) is fitted to the data. It gives averaged dwell times of $< \tau_{\mathrm{down}} >= 0.022$ s and $< \tau_{\mathrm{up}} >= 0.018$ s. (e) Influence of the bin size on the average dwell times in panels d and e. (f) Average dwell times for varying thresholds R_L and R_H. The average time $< \tau_s >$ is the time spent in between the two thresholds.

S2. Electrothermal backaction

A possible mechanism of nonlinear friction is the electrothermal backaction due to circuit retardation. The mechanism can be viewed as follows. The nanotube displacement δz affects the charge on the nanotube and thus influences the nanotube conductance G. The change of the conductance δG can be expanded in a series in δz. In turn, the conductance change induces variations in the power dissipated by the current flowing through the nanotube. Through the Joule effect, the change in power δP leads to a temperature change δT. This change can be also expanded in a series in δP. Importantly, the temperature change modifies

the mechanical tension δT_{mech}. To the leading order $\delta T_{\mathrm{mech}} \propto \delta \mathrm{T}$. The change of the tension shifts the resonance frequency $\delta\omega$, thereby affecting the nanotube displacement δz.

This backaction mechanism is mediated by the circuit, which determines the current through the nanowire. The presence of capacitance in the circuit brings delay into the backaction, giving rise to an effective friction force. The friction force can be negative. This was shown for the linear component of the friction force in Ref.[1] The nonlinear contribution, arising from higher-order terms in the expansions mentioned above, is comparatively weaker. Roughly, the magnitude of the nonlinear friction force compared to the linear one is proportional to the square of the ratio of the displacement amplitude δz to the distance to the gate electrode z_g. The corresponding nonlinear friction may still be important, as it "competes" with the generally weak nonlinear friction caused by the intrinsic mechanical nonlinearity of the nanotube. But overall, because of the aforementioned smallness, we expect the nonlinear friction due to the electrothermal backaction to be weaker than the nonlinear friction arising from the backaction, which is due to the effect of the vibrations on electron tunneling.

S3. Rotating wave approximation

This section details the derivation of Eq. 2 from the main manuscript. We assume that the mode coordinate $q(t)$ of the nanotube is described by the single-degree-of-freedom model

$$\ddot{q} + {\omega_0}^2 q + \left(2\Gamma + \tilde{\gamma}_{\mathrm{nlf}} q^2 + \Gamma_q q^4\right)\dot{q} + \tilde{\gamma}_D q^3 = 0, \tag{S1}$$

where the overdot stands for derivative with respect to time t. Furthermore, ω_0 is the radial natural frequency, $\tilde{\Gamma}$, $\tilde{\gamma}_{\mathrm{nlf}}$, Γ_q are the linear, quadratic and quintic friction coefficients, respectively. Equation (S1) includes a cubic nonlinear stiffness through the Duffing coefficient $\tilde{\gamma}_D$, that is associated with the geometric nonlinearity emerging due to the high mechanical compliance of the CNT. Next we transform Eq. (S1) from the fast-oscillating coordinate $q(t)$ to slow-time variables. To that end, we utilize the complex amplitude $\tilde{z}(t) = [q +$

$i(p/m\omega_0)]\exp(i\omega_0 t)$ and its complex conjugate $\tilde{z}^*$. Here q and p are the mode coordinate and momentum, m the effective mass, ω_0 is the eigenfrequency. The slow-dynamics in the rotating wave approximation (RWA),[2] is $q(t) = \frac{1}{2}\left(\tilde{z}(t)\exp\left(-i\omega_0 t\right) + \tilde{z}^*(t)\exp\left(i\omega_0 t\right)\right)$. We apply the method of averaging to eliminate the fast dynamics, yielding the equation for the complex amplitude $\tilde{z}$ as follows

$$\dot{\tilde{z}} = -\Gamma\tilde{z} + \left(\frac{3i\tilde{\gamma}_D}{8\omega_0} - \frac{\tilde{\gamma}_{\text{nlf}}}{8}\right)\tilde{z}^2\tilde{z}^* - \frac{\Gamma_q}{16}\tilde{z}^3\tilde{z}^{*2}. \tag{S2}$$

Now, we introduce the scaling parameters C_z for the mode, that is $z = C_z\tilde{z}$.

The coefficients of Eq. (S2) are rescaled as $\gamma_D = \frac{3\tilde{\gamma}_D}{8\omega_0 C_z^2}$, $\gamma_{\text{nlf}} = \frac{\tilde{\gamma}_{\text{nlf}}}{8C_z^2}$, and $C_z^4 = \frac{\Gamma_q}{16}$ to obtain Eq. 2 in the main manuscript

$$\dot{z} = -\left[\Gamma + (\gamma_{\text{nlf}} - i\gamma_D)|z|^2 + |z|^4\right] z. \tag{S3}$$

S4. Quintic nonlinear friction

A simple microscopic mechanism of quintic nonlinear friction used in the main text and described by the term $\propto \Gamma_q$ in Eq. (S2) is the coupling to a bath described by the Hamiltonian

$$H_q = q^3 h_{\text{b}} \tag{S4}$$

Here h_{b} is a function of the bath variables. To the lowest order in this coupling, the reaction of the bath on the vibrations can be described by the standard linear response theory,

$$\langle \delta h_{\text{b}}(t)\rangle = -\int_0^\infty dt' \mathcal{X}_{\text{b}}(t') q^3(t - t') \tag{S5}$$

where $\mathcal{X}_{\text{b}}(t)$ is the susceptibility of the bath.

It is seen from Eq. (S4) that the reaction (S5) leads to the backaction force from the

bath on the mode of the form $F_q = -3q^2(t)\langle\delta h_b(t)\rangle$. To calculate $F_q(t)$, one can substitute expression (S3) for $x(t)$ into the equation for $\langle\delta h_{\rm b}(t)\rangle$. If the Fourier transform

$$\chi_{\rm b}(\omega) = \int_0^\infty dt e^{i\omega t}\mathcal{X}_{\rm b}(t)$$

is smooth near frequency $3\omega_0$, one can then replace in Eq. (S5) $z(t-t')$ and $z^*(t-t')$ with $z(t)$ and $z(t')$, respectively [cf.[2,3]]. One then obtaines Eq. (S2) for $\dot{z}$ with the coefficient of quintic nonlinear friction Γ_q of the form

$$\Gamma_q = \frac{3}{2m\omega_0}\mathrm{Im}\,\chi_{\rm b}(3\omega_0) \tag{S6}$$

We note that the coupling (S4) also leads to a quintic nonlinear restoring force, which would be described by the term $\propto |z|^4 z$ in Eq. 2 of the main manuscript. However, this term does not lead to qualitatively new results compared to the Duffing nonlinearity in the range of amplitudes we consider. The quintic nonlinear friction is needed because the coefficients of linear and standard nonlinear friction Γ and $\gamma_{\rm nlf}$ are small in the absolute values in the region of coexistence of the quiet state and the state of self-sustained vibrations. We also note that quintic nonlinear friction can come from lower-order in x nonlinear coupling to the bath taken to a higher order of the perturbation theory and from a nonlinear response of the bath to such coupling.

In quantum terms, the terms in the considered coupling that are responsible for the quintic nonlinear friction have the form $H_q = (\hbar/2m\omega_0)^{3/2}(a^3 + a^{\dagger 3})h_q$, where a and $a^\dagger$ are the ladder operators of the oscillator. They describe relaxation processes in which the oscillator goes over 3 energy levels with the energy $\approx 3\hbar\omega_0$ transferred to the thermal bath, as indicated in the main text.

S5. Stochastic dynamics of the nanotube

In this section, we detail out the numerical simulations of the fluctuation dynamics of the nanotube, as presented in Fig. 4 of the manuscript. Our objective is to replicate the experimental findings and qualitatively match the evolution of the slow dynamics (see Fig. 1 in the main text). To that end, we simulate the quadratures (X,Y) that correspond to the real and imaginary part of z as governed by Eq. (S3) (cf. Eq. 2 of the main manuscript). By separating the imaginary and real components, we obtain the dimensionless equations:

$$\begin{cases} \dot{\mathrm{X}} = -\left(\Gamma \mathrm{X} + (\gamma_{\mathrm{nlf}}\mathrm{X} + \gamma_D \mathrm{Y})\left(\mathrm{X}^2+\mathrm{Y}^2\right) + \mathrm{X}\left(\mathrm{X}^2+\mathrm{Y}^2\right)^2\right) \\ \dot{\mathrm{Y}} = -\left(\Gamma \mathrm{Y} + (\gamma_{\mathrm{nlf}}\mathrm{Y} - \gamma_D \mathrm{X})\left(\mathrm{X}^2+\mathrm{Y}^2\right) + \mathrm{Y}\left(\mathrm{X}^2+\mathrm{Y}^2\right)^2\right) \end{cases} \tag{S7}$$

As the central point of our work is that the hysteresis-free bistability is revealed through the presence of noise, we focus on the stochastic dynamics. To that end, we incorporate noise into the above equations of motion and write stochastic differential equations:

$$\begin{cases} d\mathrm{X} = -\left(\Gamma \mathrm{X} + (\gamma_{\mathrm{nlf}}\mathrm{X} + \gamma_D \mathrm{Y})\left(\mathrm{X}^2+\mathrm{Y}^2\right) + \mathrm{X}\left(\mathrm{X}^2+\mathrm{Y}^2\right)^2\right) dt + \sigma dW_1 \\ d\mathrm{Y} = -\left(\Gamma \mathrm{Y} + (\gamma_{\mathrm{nlf}}\mathrm{Y} - \gamma_D \mathrm{X})\left(\mathrm{X}^2+\mathrm{Y}^2\right) + \mathrm{Y}\left(\mathrm{X}^2+\mathrm{Y}^2\right)^2\right) dt + \sigma dW_2 \end{cases} \tag{S8}$$

in which $W_1(t)$ and $W_2(t)$ are independent Wiener processes. These processes are constructed as normally distributed random variables with a mean of zero and a variance of dt. The asymptotic independence of these processes in the rotating frame can be traced back to the untranslated work of Bogoliubov done in 1940s, see also.[2]

To capture the complex variability of the amplitude observed within the instability region (Fig. 1(a) of the main text), we expand $\gamma_{\mathrm{nlf}} = \sum_{n=0}^{4} \gamma_n V_{\mathrm{sd}}^n$. The values of γ_n are determined

by solving the steady state dynamics of Eq. (S3), $\dot{z} = 0$, such that the nontrivial solution, i.e. the amplitude of the self-oscillatory state, matches the experimental datapoints. This is done by building a system of algebraic equations in the γ_n coefficients while imposing constraints to the vibration amplitude $\sqrt{\mathrm{X}^2 + \mathrm{Y}^2}$. Following the experimental observation, we adjust the isolated branch such that the saddle node bifurcation points, responsible for the initialization and termination of the isola, align with the boundaries of the instability region, i.e. V_{sd} =0.2 and 0.4 mV. We determine the higher-order coefficients of γ_{nlf} in Table 1. Additionally, to mimic the complex behavior of the vibration amplitude within the bistable

Table 1: Coefficients for the higher-order quadratic non-monotonic damping coefficient.

γ_0	γ_1	γ_2	γ_3	γ_4
2336.517	-31737.724	157406.651	-339858.316	270446.292

region, as shown in Fig. 4 of the main manuscript, we employ the coefficients $\Gamma = 1.25$ and $\gamma_D = 0.2$. The system in Eq. (S8) with the set parameters is numerically integrated using the Euler-Maruyama method with $\sigma = 0.6$, to obtain a long-time realization (t $= 10^6$ steps with $dt = 0.005$) of the stochastic dynamics of the nanotube. The result of the time integration is what is shown in Fig. 4 of the main text where we study vibrational bistability revealed by noise (scaling parameter $C_z = 1\mathrm{nm}^{-1}\mathrm{s}^{-1/4}$).

References

(1) Urgell, C.; Yang, W.; De Bonis, S. L.; Samanta, C.; Esplandiu, M. J.; Dong, Q.; Jin, Y.; Bachtold, A. Cooling and self-oscillation in a nanotube electromechanical resonator. *Nature Physics* **2020**, *16*, 32–37.

(2) Bachtold, A.; Moser, J.; Dykman, M. I. Mesoscopic Physics of Nanomechanical Systems. *Rev. Mod. Phys.* **2022**, *94*, 045005.

(3) Dykman, M. I.; Krivoglaz, M. A. Classical theory of nonlinear oscillators interacting with a medium. *physica status solidi (b)* **1971**, *48*, 497–512.
